# Retraction Note: Gender-specific determinants of overweight and obesity among older adults in India: evidence from a cross-sectional survey, 2017-18

**DOI:** 10.1186/s12889-024-20482-0

**Published:** 2024-10-23

**Authors:** Amiya Saha, Bittu Mandal, T. Muhammad, Papai Barman, Waquar Ahmed

**Affiliations:** 1 Department of Family & Generations, International Institute for Population Sciences, Mumbai, 400088 India; 2 School of Humanities and Social Sciences, Indian Institute of Technology, Indore, 453552 India; 3 Tata Institute of Social Sciences, Mumbai, 400088 India

**Retraction Note: BMC Public Health (2023) 23:2313**

10.1186/s12889-023-17156-8

The Editor has retracted this article because of substantial overlap with previously-published papers by some of the same authors [1, 2]. Amiya Saha does not agree to this retraction. Bittu Mandal, T. Muhammad, Papai Barman and Waquar Ahmed did not respond to correspondence regarding this retraction.
